# Characterization of the enzymatic activity of the serine protease domain of Factor VII activating protease (FSAP)

**DOI:** 10.1038/s41598-019-55531-x

**Published:** 2019-12-12

**Authors:** Nis V. Nielsen, Elfie Roedel, Dipankar Manna, Michael Etscheid, Jens Preben Morth, Sandip M. Kanse

**Affiliations:** 1 Oslo University Hospital and University of Oslo, Oslo, Norway; 2 Justus Liebig University, Giessen, Germany; 3 Paul Ehrlich Institute, Langen, Germany; 4 Norwegian Center of Molecular Medicine, Nordic EMBL Partnership University of Oslo, Oslo, Norway

**Keywords:** Proteases, Proteases, Molecular modelling, Molecular medicine

## Abstract

Factor VII (FVII) activating protease (FSAP) is a circulating serine protease. Human genetic studies, based on the Marburg I (MI) (Gly221Glu, chymotrypsin numbering system) polymorphism, implicate FSAP in the pathogenesis of many diseases. Here, we describe the molecular and functional changes caused by the Gly221Glu substitution in the 220 loop using recombinant proteins expressed in *E. coli*. The serine protease domain (SPD) of wild type (WT) FSAP displayed auto-catalytic activation whereas the MI isoform displayed very low autocatalytic activation and low proteolytic activity against the chromogenic substrate S-2288, Factor VII, tissue factor pathway inhibitor as well as pro-urokinase. Introduction of a thermolysin cleavage site in the activation position (Arg15Gln) led to cleavage of both WT- and MI-SPD and the resulting WT-SPD, but not the MI-SPD, was active. Mutating the Gly221 position to Asp, Gln and Leu led to a loss of activity whereas the Ala substitution was partially active. These results suggest a disturbance of the active site, or non-accessibility of the substrate to the active site in MI-SPD. With respect to regulation with metal ions, calcium, more than sodium, increased the enzymatic activity of WT-SPD. Thus, we describe a novel method for the production of recombinant FSAP-SPD to understand the role of the MI-single nucleotide polymorphism (SNP) in the regulation of its activity.

## Introduction

**Figure 1:**
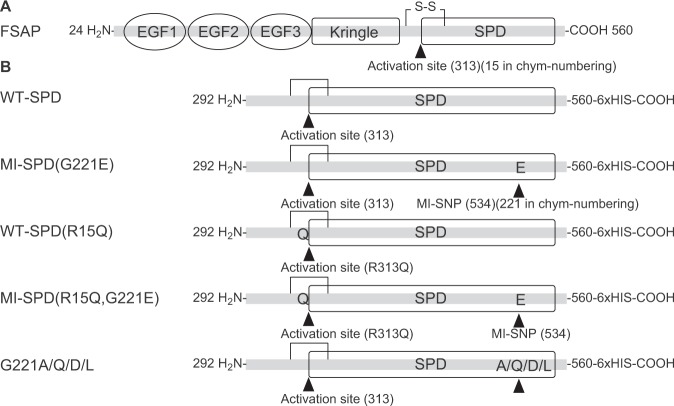
Schematic structure of FSAP and the proteins expressed. (**A**) FSAP has 3 EGF domains, a kringle domain followed by a catalytic serine protease domain (SPD). (**B**) Schematic structure of the different constructs expressed in this study. Arrows indicates the position of the activation site (313) and the position of the MI-polymorphism (534) as well as their position in the chymotrypsin numbering system.

Factor VII (FVII) activating protease (FSAP) is a circulating serine protease produced in hepatocytes; the initial gene product is secreted as an inactive zymogen called pro-FASP^1^. The full-length protein includes three epidermal growth factor (EGF) domains followed by a kringle domain and a C-terminal serine protease domain (SPD)^2^ that is homologous to the SPD domains found in the hepatocyte growth factor activator (HGFA), Factor XIIa as well as tissue and urokinase plasminogen activator (tPA and uPA) (Fig. 1A). Pro-FSAP interacts with positively or negatively charged template molecules and undergoes a conformational change leading to autocatalytic activation^3^. Cleavage of pro-FSAP at position Arg15-Ile16 between the SPD in the light chain, and the heavy chain^4^, is presumed to, expose a new N-terminus (Ile16) which forms a salt-bridge with Asp194 as in other members of the chymotrypsin family^5^ (chymotrypsin numbering system). Based on homology to other coagulation factors it has been proposed that^6^, FSAP has a 70-loop which binds Ca^2+^ and a 220-loop/180-loop^7^ which binds Na^+^.

About 5% of the Caucasian population are carriers of a single nucleotide polymorphism (SNP) in the FSAP gene, called the Marburg I (MI) SNP, that results in an exchange of a single amino acid (Gly221Glu) in the protease domain^8^. The amino acid alteration is in the 220-loop that is close to the S1 pocket of the active site. MI-FSAP, isolated from human plasma, has about 5-fold lower proteolytic activity than the WT counterpart against various substrates such as pro-uPA, factor VII (FVII), tissue factor pathway inhibitor (TFPI) and kininogen^9^. The MI-SNP was found to be linked to late complications of carotid stenosis^10^, liver fibrosis^11^, thyroid cancer^12^ and stroke^13^. When tested in disease model systems, FSAP^−/−^ mice have more severe liver fibrosis^14^, stroke^15^, neointima formation^16^ and are protected against thrombosis^17^. Thus, it is pertinent to understand why MI-FSAP has lower enzymatic activity and how this leads to patho-physiological consequences.

Recombinant FSAP was produced in mammalian cells by three groups to date^18–20^. Overexpression of WT-FSAP induced cell-death by apoptosis thus, limiting expression. This problem could be overcome by introducing point mutations that inactivated FSAP^18^. Hasumi and co-workers used a similar strategy to produce catalytically inactive Ser195Ala mutant for use in binding studies^20^, while Mertens and co-workers solved this problem by introducing a mutation at the activation site (Arg15Gln) making it resistant to auto-activation. The Arg15Gln mutant remained proteolytically inactive until cleaved by exogenously added thermolysin^19^. They suggest that the Gly221Glu polymorphism disturbs the transition of the zymogen form into a catalytically active protease^7^. Thermolysin is a common zinc dependent protease that cleaves at positions followed by hydrophobic residues. The problem with thermolysin is that it can potentially cleave FSAP at >100 different sites, introducing potential artefacts in downstream experiments. Thus, there is a need to establish a robust expression system for recombinant active FSAP.

Here we describe a novel strategy to express the serine protease domain (SPD) of FSAP in *E. coli* and perform a biochemical characterization of the wild type (WT)-SPD and the MI-SPD isoform against natural and chromogenic substrates and study their regulation by inhibitors and metal ions. Additional mutations were introduced in the MI position to understand the reasons for the loss of activity. Binding of active site ligands and homology modelling was performed to characterize these isoforms.

## Materials and Methods

### Expression of recombinant SPD-FSAP

Full length FSAP, excluding the signal peptide, (*amino acids 24–*560*)* (numbering system *in italics* refers to complete FSAP including the signal peptide), SPD-FSAP (amino acids *292–560*) and various other mutants were cloned into the pASK-IBA33plus vector (IBA-Lifesciences, Goettingen, Germany) including a C-terminal 6 X His tag. SPD-FSAP contains 22 amino acids from the C-terminal of the heavy chain in addition to the complete light chain, protease domain, including the inter-chain disulphide bond. Additionally, a Met residue was added at the N-terminal end and Ser-Ala-Arg-Gly-Ser sequence was added before the His tag. All mutants were prepared by site directed mutagenesis (Stratagene, La Jolla, CA) (Fig. 1B). Positively transfected BL21-Gold cells (DE3) (Agilent, Santa Clara, CA) were selected on 100 µg/ml ampicillin agar plates. Anhydro-tetracycline 300 µg/L was used for the induction of expression and inclusion bodies were prepared. The inclusion bodies were washed by centrifugation and sonicated in 50 mM Tris (pH 8.0), 5% (vol/vol) glycerol, 100 mM NaCl, 1 mM 2-mercaptoethanol, 100 µg/ml of lysozyme and EDTA-free protease inhibitor tablets (Roche, Mannheim, Germany) and pelleted by centrifugation. Inclusion bodies were resuspended in TNU buffer (100 mM Tris pH 8.0, 300 mM NaCl, 5 mM 2-Mercaptoethanol, 8 M Urea,) for 1 h. The soluble fraction was loaded onto pre-equilibrated Ni-Agarose column (Qiagen, Hilden, Germany) and washed with TNU buffer with 15 mM imidazole, and eluted with TNU buffer containing 400 mM imidazole.

### Refolding

Refolding was monitored and optimized by determining the hydrolysis of the chromogenic substrate S-2288 as described later. The unfolded/purified FSAP was added dropwise to 100 mM Tris (pH 7.5), 125 mM MgCl_2_, 100 mM KCl, 2 mM reduced Glutathione (GSH), 1 mM oxidized Glutathione (GSSG), 1 M NDSB-201 (3-(1-Pyridinio)-1-propanesulfonate), 5% glycerol at 21 °C at a ratio of 20 mg protein per 100 ml buffer. The preparation was mixed slowly for 48 h followed by centrifugation at 12,000 × *g* for 10 min to clear the precipitate. FSAP-SPD was further concentrated using spin concentrator and re-purified on a Superdex 75 column (GE Healthcare, Oslo, Norway) in gel filtration buffer (10 mM Tris pH 8.0, 150 mM NaCl) using an ÄKTA Purifier (GE Healthcare). Recombinant proteins were characterized by N-terminal sequencing using the Edman procedure (Guenther Lochnit, University of Giessen, Germany). After gel-filtration the preparation of WT-SPD was prone to auto-proteolysis upon storage and it was used immediately in experiments.

### Active site titration and kinetic analysis of FSAP

Active site titration was performed as described earlier^21^. The enzyme was buffer-exchanged into 5 mM Tris (pH 8.0), 150 mM NaCl, 2 mM CaCl_2_. 50 µM of *p*-nitrophenyl *p*’-guanidinobenzoate (PNPGB) in methanol was used as substrate and the burst was measured at 402 nm using 18300 M^−1^ cm^−1^ as extinction coefficient. FSAP activity assays were performed as described previously^22^. In brief, 96 well microtiter plates were used and the standard assay system consisted of 25 mM Tris (pH 7.4), 137 mM NaCl, with CaCl_2_ (2 mM) and 0.1% (wt/vol) Tween-20 and 0.25 mM of the chromogenic substrate S-2288 (H-D-isoleucyl-L-prolyl-L-arginine-p-nitroanilinedihydro-chloride) (Chromogenix, Mölndal, Sweden) and was followed over a period of 60 min at 37 °C at 405 nm in a microplate reader. The data was fitted to the Michaelis-Menten equation using Graphpad Prism software, Version 7.02 (San Diego, CA).

### Activation of Pro-uPA and FVII

FSAP-SPD was incubated with pro-uPA (Grünenthal, Stolberg, Germany) and its activation was measured by adding 0.2 mM of the chromogenic substrate S-2444 (L-pyroglutamyl-glycyl-L-arginine-p-nitroanilinedihydro-chloride) (Chromogenix). Absorbance was followed for 60 min at 37 °C at 405 nm in a microplate reader. FVIIa clotting activity was determined in the presence of a recombinant soluble mutant tissue factor (TF)^23^. Briefly, FVIIa solutions were mixed with equal volumes of FVII-deficient plasma (Roche Diagnostics, Mannheim, Germany), a mixture of TF and phospholipids (PTT reagent, Diagnostica Stago, Asnieres, France) and 25 mM CaCl_2_. Clotting and turbidity changes were monitored at 37 °C and 405 nm in a microplate reader. In each well the final concentration of total FVII/FVIIa was set to 5 nM and TF to 60 nM. The level of FVIIa generated was quantified relative to the WHO 1^st^ International Standard FVIIa concentrate (1 IU/ml FVIIa corresponds to 20 ng/ml or 0.5 nM).

### Inactivation of TFPI

Recombinant full length TFPI (provided by T. Hackeng, Maastricht, The Netherlands) was incubated at 37 °C for 30 min with 25–200 nM WT- and MI-SPD, respectively, in HBS-BC (HEPES (25 mM), pH 7.4, NaCl (100 mM) supplemented with bovine serum albumin (BSA) (0.5% wt/vol) and CaCl_2_ (3 mM). The reaction was stopped by transferring 10 µl of the reaction mixture to 1 µl of 160 µM aprotinin. The residual TFPI activity was measured as described previously with minor modifications^24^. In brief, 90 µl of 0.44 nM FXa (Haematologic Technologies Inc, Essex Junction, VT) diluted in HBS-BC + 0.1% wt/vol Tween-20) was added to the aprotinin-treated reaction mixture and was incubated for 15 min at 37 °C to allow complex formation between TFPI and FXa. After addition of 40 µl of pre-warmed phospholipids (PTT reagent) and 20 µl of 2 mM chromogenic substrate S-2765 (N-a-Benzyloxycarbonyl-D-arginyl-L-glycyl-L-arginine-p-nitroaniline-dihydrochloride) (Chromogenix), the residual FXa activity was measured at 405 nm and 37 °C in a plate reader. Residual TFPI activity was quantified relative to a dilution series of TFPI incubated with FXa as described above.

### Binding of biotinylated D-phenylalanyl-prolyl-arginyl chloromethyl ketone (Biot-PPACK)

High binding ELISA plates (Costar, Cambridge, MA) were coated with polyclonal rabbit FSAP antibody (5 ug/ml) and after blocking with BSA (3% wt/vol), WT- or MI-SPD was captured. Biot-PPACK, Biot-aprotinin, Biot-Mab570 was added in a buffer with 25 mM Tris (pH 7.4), 137 mM NaCl, with 2 mM CaCl_2_, 0.3% (wt/vol) BSA and 0.1% (wt/vol) Tween-20. After extensive washing the plates were developed with peroxidase coupled streptavidin and developed with a 3,3′,5,5′-tetramethylbenzidine (TMB) substrate. Binding to plates without the addition of WT- or MI-SPD was used to obtain blank values, which were subtracted from the binding in the presence of the SPD’s to obtain specific binding. In competition experiments the binding of Biot-PPACK was determined in the presence of increasing concentrations of aprotinin or PPACK.

### Homology modelling of WT- and MI-SPD

The homology models of the FSAP-SPD were generated using the protein structure prediction programme I-TASSER^25^. The 247 residues at the C-terminus (Ile314-Phe560) were used as the input sequence, and the models were built using uPA (PDB ID: 2VNT) as a template. All models yielded a C-score (confidence score) in the range of 0.83–0.85 and a TM-score (structural similarity) of 0.83 ± 0.08, indicating the correct topology and similarity of the models^26^. For the analysis of the metal ion binding sites, the WT-SPD model was superimposed on FXa structure (PDB ID: 2JKH), where the Na^+^ and Ca^2+^ binding sites are described. The models were visualized and the figures were prepared by using PyMOL Molecular Graphics System; Version 1.7.2.0 (Schrödinger, LLC).

## Results

### Expression and characterization of FSAP-SPD

Full-length FSAP (*amino acids 24–560*) was expressed in *E. coli* but could not be refolded from inclusion bodies. A SPD construct spanning a 22 amino acids of the heavy chain (*amino acids 292*–*560*) was produced effectively in inclusion bodies and could be refolded into the active protease (see Fig. 1B for details of the constructs used in this study) and used for all the experiments in this study. The inter-chain disulphide bond between the heavy chain and Cys122 in the protease domain is retained in this construct. The refolding conditions were optimized by measuring the hydrolysis of the chromogenic substrate S-2288. The activity status of this preparation indicated that the protein underwent classical autocatalytic activation with cleavage at Arg15-Ile16 during the refolding process (see later). The WT-SPD was very stable in refolding buffer. Based on the amount of input protein, the overall yield from the refolding process was 10–15% due to extensive precipitation. Active site titration showed that 59 ± 4% (n = 3 different preparations) of the final soluble WT-SPD preparation was active. The homogeneous size of the preparation would suggest that the inactive fraction was falsely folded but this was not investigated further. In preliminary experiments, affinity selection on benzamidine-sepharose increased the active site concentration significantly but this also enhanced auto-proteolysis. Thus, we have established a robust method for preparation of SPD of FSAP by producing it as an unfolded protein in inclusion bodies and generating the active form by controlled refolding.

We performed extensive tests to check if the protein could be expressed in a soluble form under different conditions in different bacteria. This included inducing the expression at low temperatures such as 15 °C, 22 °C and 30 °C. The cell pellets were extracted with eight different solubility buffers with varying pH, chaotropic agents, and ion compositions but yielded no functional soluble protein. Supplementary Fig. S1 shows FSAP expression in different bacterial strains at different time points up to 4 h post induction.

### Autocatalytic activation of WT and MI-SPD

**Figure 2:**
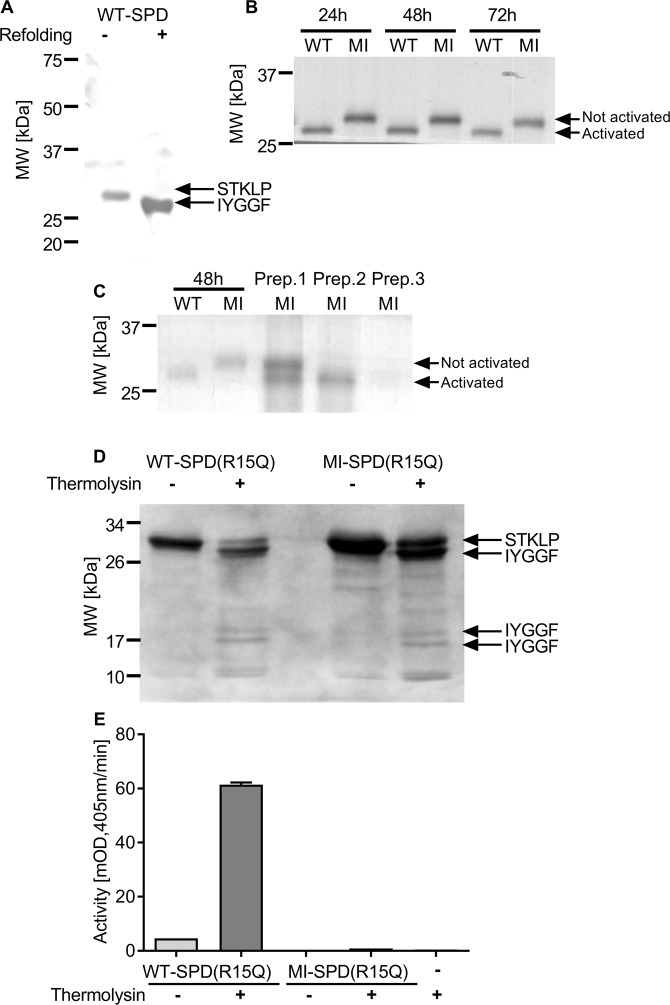
Activation of WT- and MI-SPD. (**A**) WT-SPD was refolded and compared to unfolded protein on reduced SDS-PAGE followed by Coomassie staining of the gel. N-terminal sequencing results corresponding to the bands are indicated on the right and the MW markers are indicated on the left. (**B**) Time course of refolding of a preparation of WT- and MI-SPD over 24–72 h. (**C**) Refolding of a preparation of WT- and MI-SPD over 48 h compared to the refolded state of 3 different preparations of MI-SPD that, after 6 months storage at −20 °C, show different degrees of activation. (**D**) WT-SPD (Arg15Gln) and MI-SPD (Arg15Gln) (5 μg) were incubated with thermolysin (1 μg/ml) for 15 min at 37 °C. SDS-PAGE followed by Coomassie staining of the gel. (**E**) The same mixture was incubated with the chromogenic substrate S-2288 and substrate hydrolysis was followed by measuring absorbance at 405 nm and represented as mOD/min (mean ± SD).

Auto-activation of WT-SPD could be followed by SDS-PAGE under reducing conditions since there would be a loss of 22 amino acids of the heavy chain and a shift in MW from 30.6 to 28.1 kDa. N-terminal sequencing of the 30.6 kDa band showed an expected sequence of STKLP and the 28.1 kDa band started with Ile16 (IYGGF) in WT-SPD (Fig. 2A). The N-terminal Met residue is removed, presumably by *E. coli* aminopeptidases. The kinetics of auto-activation of WT-SPD showed a maximal activation at 48 h at 4 °C (Fig. 2B). MI-SPD showed no activation for up to 3 days (Fig. 2B), and in further experiments for up to 1 week, but prolonged storage at 4 °C for months lead to auto-activation in some preparations (Fig. 2C). Preparations of MI-SPD showing auto-activation, by a shift in MW, had very low enzymatic activity against the chromogenic substrate S-2288. In the refolding step the recovery of MI-SPD, with respect to protein amount, was about 2-fold higher than WT-SPD. Because of its very low catalytic activity it was not possible to perform active site titration on MI-SPD.

We also compared auto-activation of WT- and MI-SPD after mutating the activation site from Arg15 to Gln to prevent auto-activation and enable controlled activation by thermolysin. The Arg15Gln mutants of both, WT and MI, isoforms showed no auto-activation, as was expected. Both could be activated by thermolysin, as confirmed by a shift in the MW of the bands as well as N-terminal sequencing (Fig. 2D). Thermolysin also cleaved the SPDs non-specifically as seen by the generation of many low MW bands starting with the original N-terminal sequence (STKLP) (Fig. 2D). The Arg15Gln mutant activated with thermolysin showed robust activity against S-2288, whereas similarly activated MI isoform showed no detectable activity (Fig. 2E). The fact that the Arg15Gln mutant folded correctly in its zymogen form suggests that the activation is not a prerequisite for the correct folding of SPD’s. Thus, the low enzymatic activity was an intrinsic property of MI-SPD and not attributed to the lack of refolding.

### Comparison of WT- and MI-SPD against physiological macromolecular substrates

**Figure 3:**
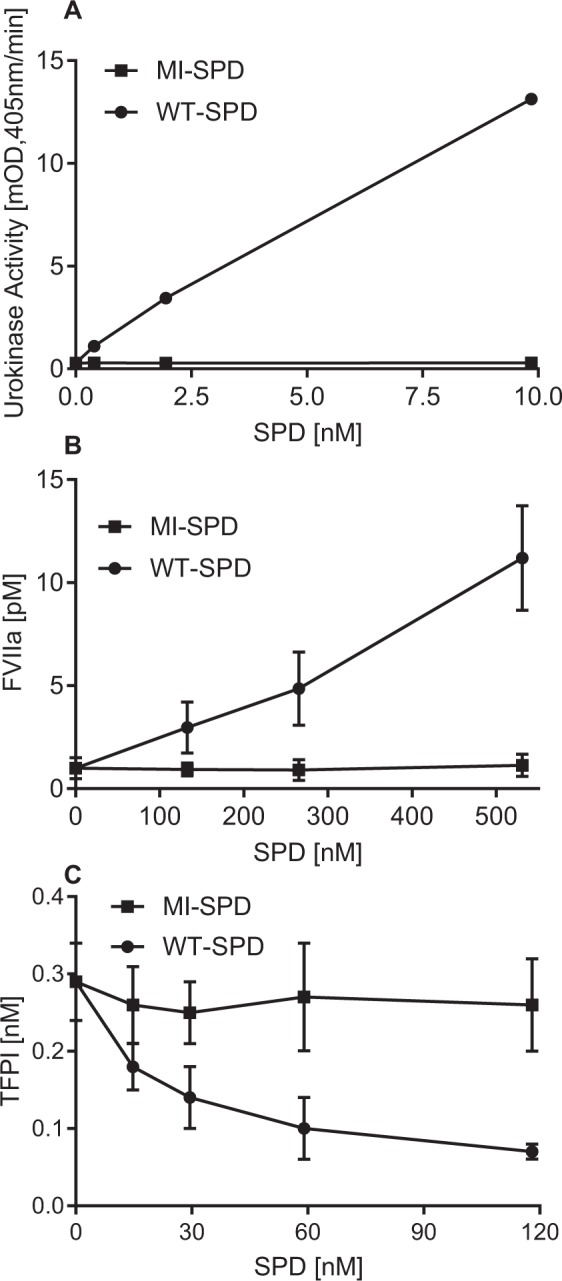
Effect of WT- and MI-SPD on physiological substrates. (**A**) Activation of pro-uPA (10 μg/ml) by WT-SPD (●) and MI-SPD (■) was performed for 15 min at 37 °C. uPA activity was measured using the hydrolysis of substrate S-2444 and is given as mean ± SD, mOD/min. The activity of SPD, alone, against S-2444, was found to be negligible. (**B**) Activation of FVII by WT-SPD (●) and MI-SPD (■) during 15 min at 37 °C. The FVIIa generated was quantified measured as described in materials and methods. (**C**) Inactivation of TFPI by WT-SPD (●) and MI-SPD (■) was performed followed by measurement of TFPI activity using the FXa inhibition assay as described in materials and methods. Results are given as nM TFPI (mean ± SD, n = 5).

Since plasma-purified FSAP has been shown to activate pro-uPA and Factor VII (FVII) as well as inactivate TFPI, we tested these natural substrates with the activated forms of WT-SPD and MI-SPD. WT-SPD was effective in activating pro-uPA as well as FVII, whereas MI-SPD had no such activity (Fig. 3A,B). FVII activation required approximately 100-fold higher concentrations of WT-SPD than pro-uPA activation, which is similar to the earlier observations with plasma-purified proteins^9^. Inactivation of TFPI was also observed with WT- but not MI-SPD (Fig. 3C) as was the case with plasma-purified FSAP^27^. Thus, the comparison of properties of WT- and MI-SPD against physiological substrates showed the expected pattern of activities. Thus, the recombinant SPD’s, even though they lack the regulatory domains, can phenocopy some of the known functions of full-length FSAP.

### Comparison of mutants at the position of MI-SNP

**Figure 4:**
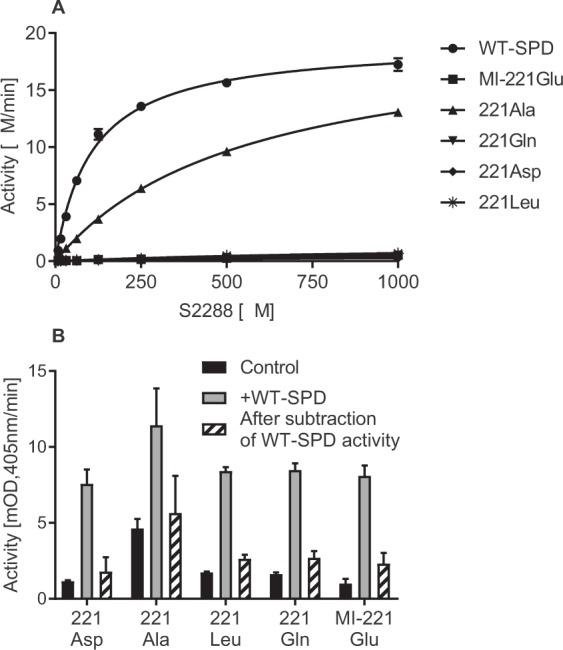
Functional effect of different amino acid replacements at the MI-position. (**A**) Michaelis-Menten plots of WT-SPD (●), MI-SPD (■), -Ala (▲), -Gln (▼) –Asp (◆) and –Leu (∗) mutants using S-2288 as a substrate. Concentration of WT-SPD was 30 nM whereas that of mutants was 300 nM and activity is given in μM/min (mean + SD). (**B)** WT- and mutant-SPD’s were refolded for 48 h and the activity against the hydrolysis of S-2288 was determined. MI- and mutant-SPD’s (5 μg/ml) were preincubated with WT-SPD (0.1 μg/ml) for 30 min at 37 °C before determining enzyme activity against the chromogenic substrate S-2288. Substrate hydrolysis was followed by measuring absorbance at 405 nm and is represented as mOD/min (mean + SD).

To elucidate the basis of the low catalytic activity of the MI-isoform (Gly221Glu) and thus test the significance of Glu in position 221, this residue was mutated to the following amino acids; Ala, Gln, Leu or Asp. Ala would represent a small amino acid that does not have the flexibility around the psi/phi torsion angles as compared to Gly. Gln has a similar size as Glu but is uncharged, however is still able to form hydrogen bonds, while Leu is similar in size, but hydrophobic and does not form hydrogen bonds. Asp is smaller than Glu but carries the same charge. These studies were performed under optimized concentrations of Ca^2+^ and Na^+^ as described later. The kinetic properties were investigated against a standard chromogenic substrate S-2288. For WT-SPD a *K*_*m*_ 107 ± 7 µM and *k*_*cat*_ of 650 ± 28 min^−1^ (n = 3) was measured. The Ala mutant had a 50-fold and the MI-mutant had a 2000-fold lower catalytic efficiency than WT-SPD. The Gln-, Leu- and Asp- mutants exhibited negligible activity (Fig. 4A, Table 1). These three mutants that exhibited negligible activity, most likely, also did not undergo auto-activation.

**Table 1:** Michaelis-Menten kinetic constants for WT-SPD, MI-SPD (Gly534Glu) and the Gly534Ala-SPD mutation using the chromogenic substrate S-2288.

	*k*_*cat*_ (min^−1^)	*K*_*m*_ (µM)	*k*_*cat*_*/K*_*m*_ (min^−1^ μM^−1^)
WT-SPD	650 ± 28	107 ± 1	6.1 ± 0.2
Gly221Glu(MI)	5 ± 0.2	2148 ± 224	0.003 ± 0.0003
Gly221Ala	70 ± 4	556 ± 81	0.13 ± 0.01
Gly221Leu	7 ± 3	1436 ± 354	0.005 ± 0.001
Gly221Asp	4 ± 3	941 ± 501	0.006 ± 0.005
Gly221Gln	5 ± 3	653 ± 245	0.009 ± 0.008

The buffer used for these experiments was Tris (50 mM, pH 7.4) containing 2 mM CaCl_2_ and Tween-20 (0.1% wt/vol). All values are mean ± SD. Each value is obtained from 3 independent experiments.

All mutants were also incubated with active WT-SPD to reinforce activation but, this did not lead to any additional change in their enzymatic activity (Fig. 4B). Thus only the replacement of the original Gly with a similarly sized Ala led to a partial retention of enzymatic activity, whereas all other replacements resulted in the same properties as MI-SPD.

### Binding of active site ligands to activated WT- and MI-SPD

**Figure 5:**
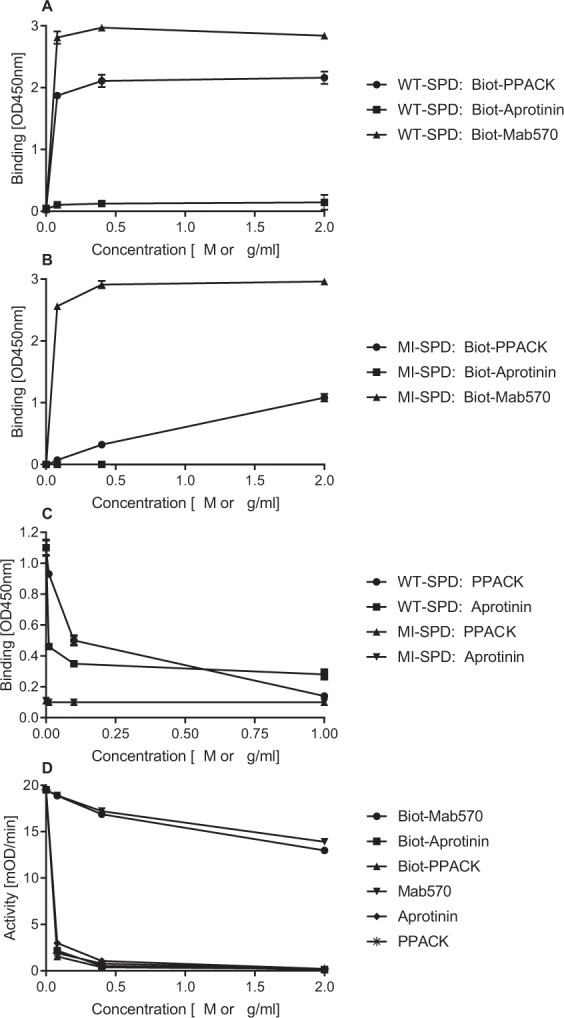
Characterization of inhibitor binding to the active site of WT-SPD and MI-SPD. (**A**) WT- or (**B**) MI-SPD (2 μg/ml) was captured on rabbit anti-FSAP polyclonal antibody-coated wells (5 μg/ml). Biot-PPACK (●, 0–2 μM), Biot-aprotinin (■, 0–2 μM) or Biot-Mab570 (▲, 0–2 μg/ml) at different concentrations was added as indicated and specific binding was determined using streptavidin POD as absorbance at 450 nm (OD, mean + SD). (**C**) In a similar experiment as in (**A**,**B**) Biot-PPACK (0.1 μM) binding to WT-SPD (●, ■) and MI-SPD (▲, ▼) was tested in the presence or absence of increasing concentrations of PPACK (●, ▲, 0–1 μM) or aprotinin (■, ▼, 0–1 μM). (**D**) The proteolytic activity of WT-SPD against S-2288 was determined in the presence of increasing concentrations of Biot-PPACK (▲) and PPACK (∗, μM), Biot-aprotinin (■) and aprotinin (◆, μM), Biot-Mab570 (●) and Mab570 (▼, μg/ml). Enzyme activity is presented as mOD/min (mean + SD). All binding and activity studies were done in the presence of 2 mM Ca^2+^ and 137 mM Na^+^.

An earlier study has suggested that after activation of the zymogen form of MI-FSAP the new N-terminus (Ile16) is unable to form a salt-bridge with Asp194 to enable a conformational change into an active protease^7^. We sought further evidence for a change in the conformation of the active site of MI-SPD by performing binding studies with biot-PPACK. These studies were performed under optimized concentrations of Ca^2+^ and Na^+^ as described later. To maintain their correct conformations, WT- and MI-SPD were captured using immobilized polyclonal FSAP antibody. Biot-PPACK bound to WT-SPD in a concentration-dependent manner but there was low binding of MI-SPD (Fig. 5A,B). An inhibitory antibody directed against the SPD of FSAP, Mab570, bound to both isoforms equally well indicating that both isoforms were captured equally well on the plate and have conserved epitopes. Biot-aprotinin showed negligible binding to both isoforms indicating that its affinity for WT-SPD is very low (Fig. 5A,B). The binding of biot-PPACK to FSAP-SPD could be competed by excess PPACK as well as aprotinin but this was not the case with MI-SPD (Fig. 5C). Thus, the low binding of biotinylated PPACK to MI-SPD is non-specific in nature. The enzymatic activity of WT-SPD was inhibited equally well by biotin-inhibitors in a comparable manner to non-biotin-inhibitors indicating that biotinylation did not change their properties (Fig. 5D). Inhibition of activity by biotinylated and non-biotinylated Mab570 at higher concentrations was identical but is not shown. Thus, WT-SPD binds to PPACK but MI-SPD does not.

### Effect of Ca^2+^ and Na^+^ on the activity of WT-SPD

**Figure 6:**
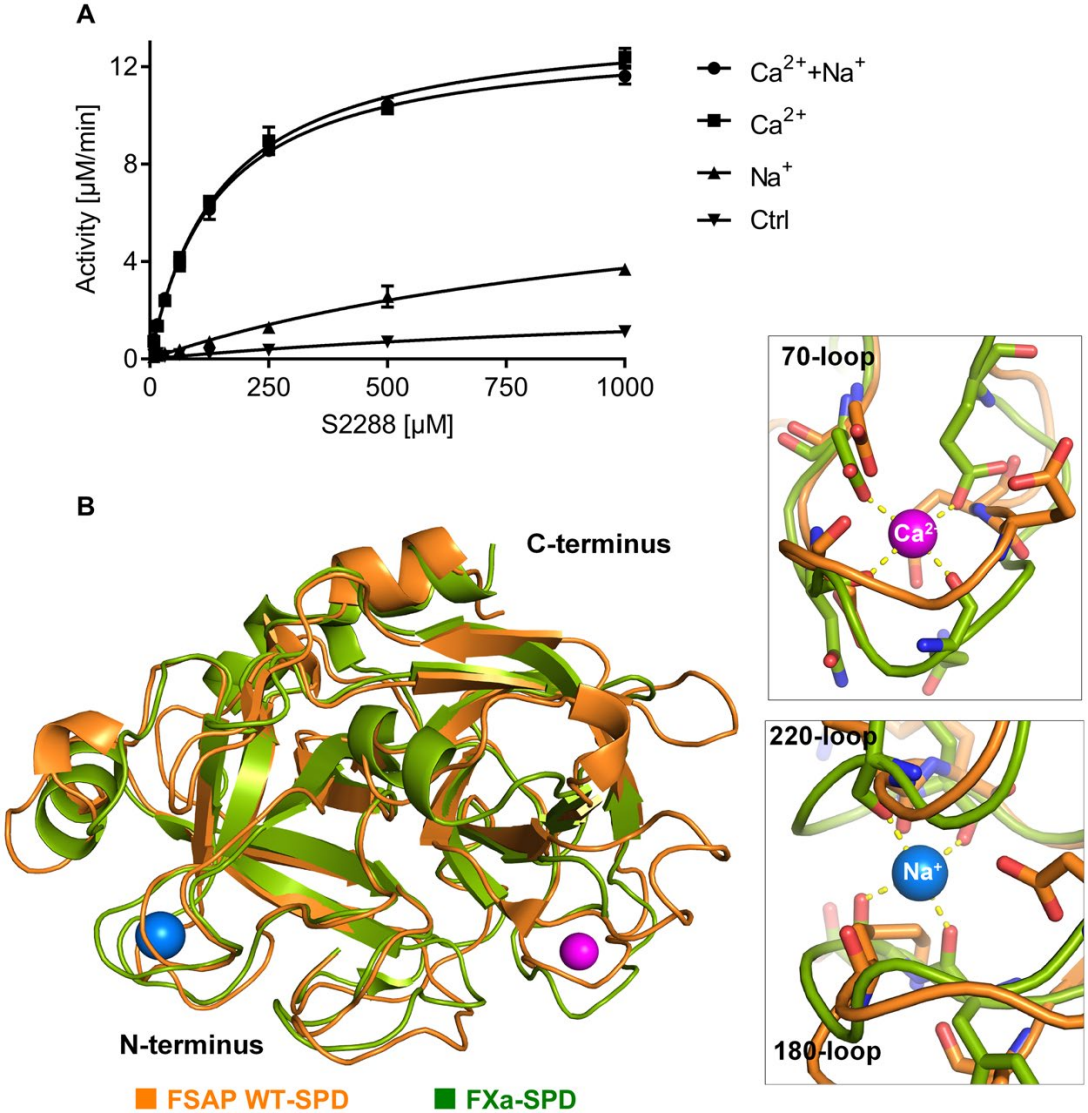
Effect of Na^+^ and Ca^2+^ on WT-SPD activity and metal ion binding sites. (**A**) The effect of Na^+^ (▲, 150 mM), Ca^2+^ (■, 5 mM), both together (●) and none (▼) on the activity of WT-SPD in the presence of increasing concentrations of the substrate S-2288 is represented as a Michaelis-Menten plot. The buffer used for these experiments was Tris (50 mM, pH 7.4) containing 2 mM EDTA and Tween-20 (0.1% wt/vol). All values are mean ± SD. (**B**) Overall structural superimposition of WT-SPD (orange) with FX (PDB ID: 2JKH, green) with cartoon presentation. The Ca^2+^ and the Na^+^ ions, taken from FX, are shown in magenta and blue spheres, respectively. The Ca^2+^ binding loop (upper inset) and the Na^+^ binding loop (lower inset) of the FX shows the corresponding amino acid residues with which they coordinate with the corresponding metal ions. The yellow dotted lines represent the hydrogen bonding.

FSAP activity has been reported^7^ to be altered through binding to the metal ions Ca^2+^ or Na^+^. We tested if physiological levels of Na^+^ and Ca^2+^ were able to modulate the activity of our WT-SPD preparation. The presence of Ca^2+^ caused a 12-fold decrease in *K*_*m*_ and a 50-fold increase in catalytic efficiency, whereas Na^+^ caused a 4-fold increase in catalytic efficiency (Fig. 6 and Table 2).

**Table 2:** Michaels-Menten kinetic constants for WT-SPD in the absence and presence of 150 mM NaCl and/or 5 mM CaCl_2_ using the chromogenic substrate S-2288.

	Buffer	Ca^2+^	Na^+^	Ca^2+^/Na^+^
*k*_*cat*_ (min^−1^)	180 ± 122	743 ± 79	458 ± 77	707 ± 61
*K*_*m*_ (µM)	1826 ± 796	146 ± 12	1275 ± 180	161 ± 27
*k*_*cat*_*/K*_*m*_ (min^−1^ μM^−1^)	0.11 ± 0.1	5.1 ± 0.9	0.4 ± 0.06	4.4 ± 0.4

The buffer used for these experiments was Tris (50 mM, pH 7.4) containing 2 mM EDTA and Tween-20 (0.1% wt/vol). All values are mean ± SD. Each value is obtained from 4–5 independent experiments.

To identify the possible metal ion binding sites in the SPD of FSAP, we superimposed the WT-SPD model with the FXa (PDB ID: 2JKH)^6^. The superimposed structures suggest the presence of both Ca^2+^ (magenta sphere) and Na^+^ (blue sphere) binding sites in WT-SPD (Fig. 6). In the WT-SPD model, compared to FXa, the amino acids Asp64, Asp66, Glu70 and Glu71 could form potential coordinates with Ca^2+^. These functional and modeling studies predict the presence of a Ca^2+^-binding site in the SPD of FSAP as described recently^7^. With regard to Na^+^, potentially the binding occurs between the residues in the 220 and 180 loops, as observed in FXa^6^. Thus, we can confirm earlier findings that Ca^2+^ is a strong activator of FSAP enzymatic activity.

## Discussion

To date the expression of recombinant active FSAP has been difficult because enzymatically active FSAP is not well-tolerated by mammalian cells^18^. One solution to this problem was to prepare a mutant that, initially, was inactive but could be activated by an exogenous protease such as thermolysin^7^. The disadvantage of this method is that the broad substrate specificity of thermolysin, which can lead to unwanted cleavage at other extraneous sites giving rise to potential artefacts. In our experiments the FSAP-SPD’s were very sensitive to multiple cleavages by thermolysin, other than at the activation site. To overcome these issues, we have developed a method to prepare recombinant SPD of FSAP in inclusion bodies of *E. coli*. The protein remains stable and inactive until it is refolded. The protein was very stable in refolding buffer at −20 °C for 1 year, or at 4 °C for several months. Most likely this buffer allows auto-activation but prevents auto-degradation due to a combination of the components of the refolding buffer and a lack of Na^+^ and Ca^2+^ that is required for full enzymatic activity. These methods will enable expression of large amounts of FSAP-SPD that is crucial for detailed biochemical, cellular and *in vivo* studies in the future.

The fact that MI-SPD showed no auto-activation for up to 1 week underscores its resistance to auto-activation. Some auto-activation was observed after months but we could not pinpoint the factors required for this in a consistent manner. Full length constructs expressed in eukaryotic cells also exhibited slower kinetics of auto-activation in MI-FSAP^7^. Mutating WT- and MI-SPD to Arg15Gln prevented auto-activation and enabled targeted cleavage by thermolysin as determined by sequencing and activity studies. Although this procedure was effective in auto-activating both WT- and MI-SPD, the activated MI-SPD remained virtually inactive. There were additional cleavages by thermolysin as seen by the appearance of a number of smaller MW bands which limits the practicality of this approach.

The MI-SPD has a reduced proteolytic activity against chromogenic substrate S-2288, FVII, pro-uPA and TFPI. Of all the amino acid replacements at the MI-position tested, only the Ala-mutant retained substantial enzymatic activity, indicating a central role of this position for the activity of the whole protein. These results are very similar to a recent report that the Ser and Ala mutants are partially active^7^ but that the MI-isoform is inactive. In concordance with the lack of enzymatic activity the binding of PPACK was also lost in MI-SPD. It has been reported that aprotinin is capable of binding to the zymogen and the active forms of some serine proteases such as trypsinogen^28^. Although aprotinin could compete with biot-PPACK for binding to the active site of WT-SPD, its affinity was too low to perform binding studies with biotinylated aprotinin under the given conditions. However, the monoclonal antibody Mab570, that inhibits the activity of WT-SPD, bound to MI-SPD equally well suggesting an overall conservation of certain epitopes in both isoforms.

In an earlier study, it has been suggested that MI-isoform is inactive because the formation of the ion-pairs between Ile16 and Asp194, which is important for achieving the zymogen-to-active conformation, is disturbed in this mutant^7^. Considering that thermolysin-cleaved WT-FSAP was compared with auto-activated MI-FSAP in these experiments there is a high potential for artefacts. Another possible explanation is that the altered flexibility in the 220-loop, when Gly is replaced by the bulkier (Leu, Gln) and negatively charged residues (Glu and Asp), interferes with substrate binding in the S1 pocket that is defined by residues 189–192, 214–212, and 224–228. Gara *et al*. used static homology models of WT- and MI-SPD and suggest differences in the conformation of the active sites between the two isoforms^12^. Further detailed crystallographic studies are needed to dissect the exact reason for the loss of enzymatic activity in MI-FSAP.

Stavenuiter *et al*.^7^ demonstrate a role for both Ca^2+^ and Na^+^ in increasing the enzymatic activity of FSAP. We found similar effects of Ca^2+^ on *K*_*m*_ and *k*_*cat*_ for WT-SPD using the substrate S-2288. Homology modeling showed a possible binding site for Ca^2+^ in the 70- and Na^+^ in the 220-loop/180-loop respectively which are in-line with the results of Stavenuiter *et al*.^7^. However, the effects of Na^+^ in our studies were much smaller. The proposed Na^+^ binding site is between 2 loops that have low sequence identity and insertions/deletions and are very flexible thus their positional assignment is uncertain.

The major advances presented in this study represent the first demonstration of the large-scale preparation of recombinant FSAP-SPD. The results obtained with the SPD-isoforms in this study largely match the earlier observations made with plasma-derived proteins^9,27^ as well as proteins obtained from mammalian expression system^7,18^. During the course of the preparation of this manuscript we have been able to develop a novel substrate, an active-site probe as well as a specific and selective inhibitor for FSAP^29^. Similarly, we also found that this preparation of FSAP can recapitulate the effects of plasma FSAP on gene expression in cells^30^. This cross-validates and further strengthens the reliability of the current approach and opens the possibility for further research in this field.

## Supplementary information

Supplementary Figure 1
