# Supplementary material for: Characterization of the enzymatic activity of the serine protease domain of Factor VII activating protease (FSAP)
Source: Sci Rep. 2019 Dec 12;9:18990. doi: 10.1038/s41598-019-55531-x (PMC6908674; doi:10.1038/s41598-019-55531-x)
Supplement: Supplementary file 1 — Supplementary Figure 1 [file 41598_2019_55531_MOESM1_ESM.pdf]

Supplementary data for:

Characterization of the enzymatic activity of the serine protease domain of Factor VII activating protease (FSAP).

Nis V. Nielsen[1], Elfie Roedel[2], Dipankar Manna[1,] Michael Etscheid[3], Jens Preben Morth[4], Sandip M. Kanse[1].

[1]Oslo University Hospital and University of Oslo; [2]Justus Liebig University, Giessen, Germany; [3]Paul Ehrlich Institute, Langen, Germany; [4]Norwegian Center of Molecular Medicine, Nordic EMBL Partnership University of Oslo, Oslo, Norway.

<u>Corresponding author:</u>

Dr. Sandip M. Kanse, Institute for Basic Medical Sciences, University of Oslo, Sognvannsveien 9, 0372 Oslo, Norway.

e-mail: sandip.kanse@medisin.uio.no

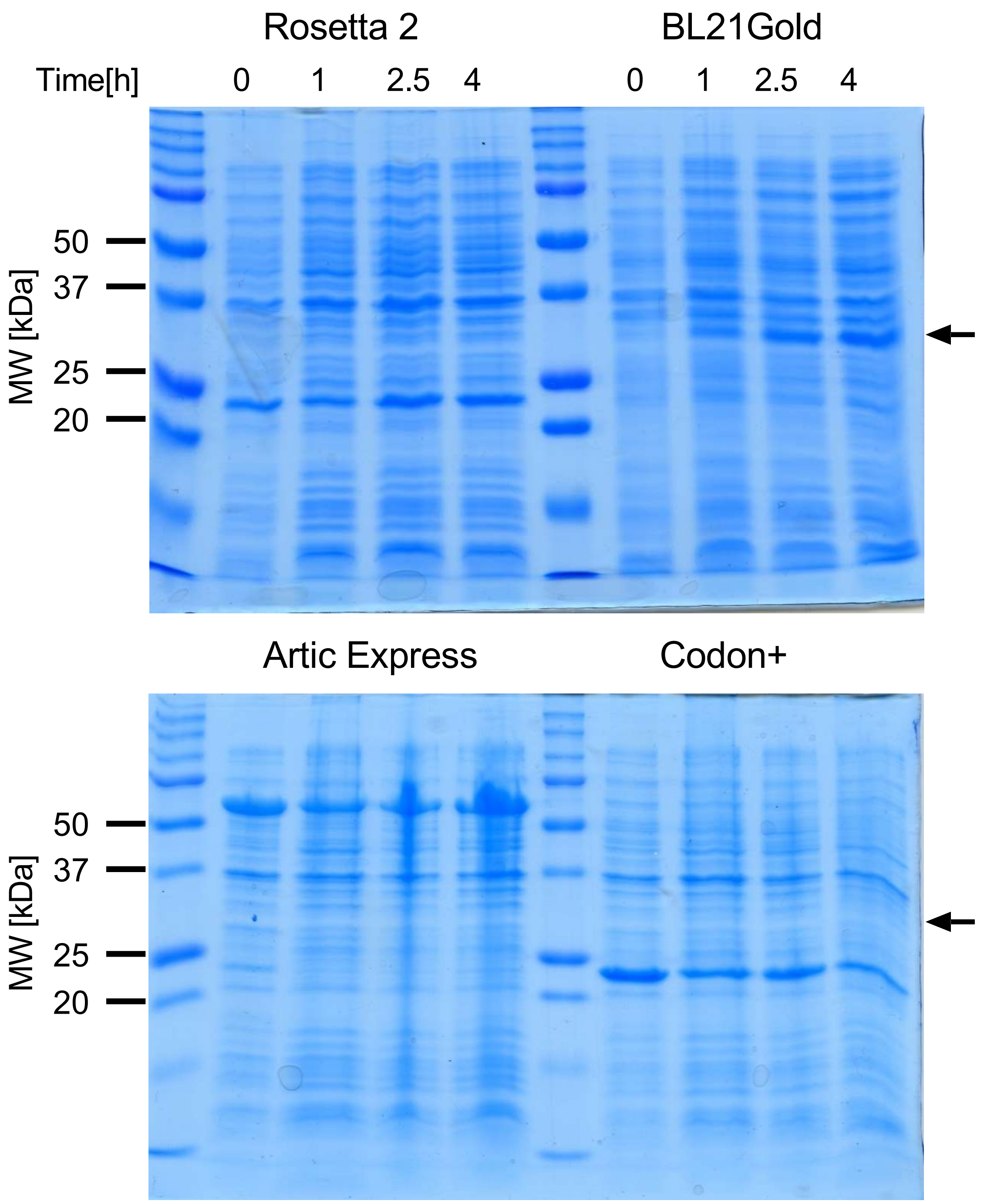

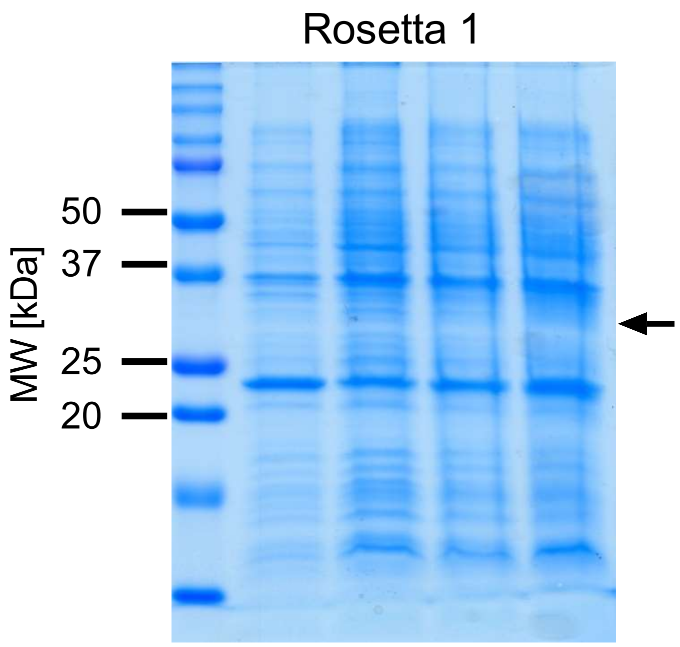

Supplemental figure 1:
SDS-PAGE analysis of the expression of WT-SPD(R15Q) in different E. coli expression strains (Artic express, BL21-Gold, BL21-Codon+, Rosetta and Rosetta2). The time indicates hours after induction with 500µg/L anhydrotetracycline hydrochloride. The arrow indicates the band corresponding to the WT-SPD(R15Q).
